# Surviving Cancer without a Broken Heart

**DOI:** 10.5041/RMMJ.10366

**Published:** 2019-04-18

**Authors:** Oren Caspi, Doron Aronson

**Affiliations:** 1 Department of Cardiology, Rambam Health Care Campus, Haifa, Israel; 2 The Ruth & Bruce Rappaport Faculty of Medicine, Technion–Israel Institute of Technology, Haifa, Israel

**Keywords:** Cancer survivorship, cancer therapy, cardio-oncology, cardiotoxicity

## Abstract

Chemotherapy-associated myocardial toxicity is increasingly recognized with the expanding armamentarium of novel chemotherapeutic agents. The onset of cardiotoxicity during cancer therapy represents a major concern and often involves clinical uncertainties and complex therapeutic decisions, reflecting a compromise between potential benefits and harm. Furthermore, the improved cancer survival has led to cardiovascular complications becoming clinically relevant, potentially contributing to premature morbidity and mortality among cancer survivors. Specific higher-risk populations of cancer patients can benefit from prevention and screening measures during the course of cancer therapies. The pathobiology of chemotherapy-induced myocardial dysfunction is complex, and the individual patient risk for heart failure entails a multifactorial interaction between the selected chemotherapeutic regimen, traditional cardiovascular risk factors, and individual susceptibility. Treatment with several specific chemotherapeutic agents, including anthracyclines, proteasome inhibitors, epidermal growth factor receptor inhibitors, vascular endothelial growth factor inhibitors, and immune checkpoint inhibitors imparts increased risk for cardiotoxicity that results from specific therapy-related mechanisms. We review the pathophysiology, risk factors, and imaging considerations as well as patient surveillance, prevention, and treatment approaches to mitigate cardiotoxicity prior, during, and after chemotherapy. The complexity of decision-making in these patients requires viable discussion and partnership between cardiologists and oncologists aiming together to eradicate cancer while preventing cardiotoxic sequelae.

## INTRODUCTION

Chemotherapy-associated myocardial toxicity is increasingly recognized with the expanding armamentarium of novel chemotherapeutic agents. The improved cancer survival has led to cardiovascular complications becoming clinically relevant many years after cancer diagnosis, and cardiovascular diseases are currently considered the main cause of death in cancer survivors. This is especially relevant for pediatric and young cancer survivors who have demonstrated increased rates of cardiovascular disease several decades after therapy.^1^,^2^ Moreover, an increasing number of patients are receiving long-term or lifelong cancer therapies with potential cardiovascular adverse effects. Thus, cardiac toxicity may lead to premature morbidity and death among cancer survivors.^3^ Beyond long-term cardiovascular complications, the onset of cardiotoxicity during cancer therapy represents a major concern and often involves clinical uncertainties and complex therapeutic decisions, reflecting a compromise between potential benefits and harm.

The pathobiology of chemotherapy-induced myocardial dysfunction is complex, and the individual patient risk for heart failure entails a multifactorial interaction among the selected chemotherapeutic regimen, traditional cardiovascular risk factors, and individual susceptibility. Treatment with several specific chemotherapeutic agents, including anthracyclines, proteasome inhibitors, epidermal growth factor receptor inhibitors, vascular endothelial growth factor inhibitors, and immune checkpoint inhibitors, imparts increased risk for cardiotoxicity that results from specific therapy-related mechanisms.

Two types of cardiomyopathy have been proposed, with anthracyclines (type I) and trastuzumab (type II) as prototypes.^4^,^5^ Type I cardiotoxicity is dose-related, associated with myocardial ultrastructural changes, and largely irreversible. Type II cardiotoxicity is not dose-related, without apparent ultrastructural abnormalities, and with high likelihood of recovery.^4^ This concept only partially describes the cardiotoxicity of anthracyclines and trastuzumab because the former can be reversible, at least in part, if detected and treated early,^6^,^7^ while recovery is not universal with the latter.^8^,^9^

## ANTHRACYCLINE-INDUCED CARDIOMYOPATHY

Anthracyclines are one of the most widely prescribed and effective cytotoxic drugs, used in the treatment of cancer. Chemotherapy regimens that include anthracycline are vastly used in the management of hematological and solid tumors, including breast cancer, lymphoma, leukemia, and sarcomas.^10^,^11^ Anthracycline-based regimens are used in approximately 1,000,000 patients annually in North America.^12^ Anthracycline-induced cardiotoxicity is the most well-studied chemotherapy-induced cardiovascular toxicity, first described in 1971.^13^

### Mechanisms of Anthracycline-induced Cardiotoxicity

The classical hypothesis for the mechanism of anthracycline-induced cardiomyopathy entails generation of excess free radicals during doxorubicin metabolism. The quinone-hydroquinone moiety of anthracyclines undergoes reduction by oxidoreductases to a doxorubicin-semiquinone radical or doxorubicinol.^14^ This quickly regenerates the parent quinone by reducing molecular oxygen to superoxide anion (O_2_^−^), and dismutation of the latter generates hydrogen peroxide (H_2_O_2_).^15^ In the presence of iron, Fe^3+^–anthracycline complexes are formed, which further catalyze the conversion of H_2_O_2_ to other reactive oxygen species (ROS), including toxic hydroxyl radicals (OH^−^; Fenton’s reaction). Conditions leading to higher iron tissue concentrations such as heterozygosity for C282Y, the hereditary hemochromatosis gene, may favor the development of doxorubicin cardiotoxicity.^16^

The cardiac mitochondria are key intracellular targets for anthracyclines, both as sites of generation of free radical intermediates, and the mitochondrial-localized production of ROS can induce mitochondrial DNA mutation and disruption of bioenergetics.^17^,^18^ Reactive oxygen species-producing enzymes such as NAD(P)H oxidase are localized in the mitochondria.

Doxorubicin (DOX) alters iron trafficking inside the cell by increasing iron incorporation into ferritin and suppressing iron release from cellular stores.^19^ Furthermore, doxorubicin leads to preferential accumulation of iron in the mitochondria, by suppressing expression of ABC protein-B8 transporter, functioning in iron export out of the mitochondria.^20^

Doxorubicin exerts its tumoricidal activity via binding to topoisomerase-IIα (Top2α),^21^ an enzyme found predominantly in dividing cells and required for DNA replication. Doxorubicin binds both DNA and Top2 to form the ternary Top2-doxorubicin-DNA cleavage complex, with subsequent blockage of DNA resealing during cell replication.

More recently, Top2β (the isoform expressed in quiescent cells including cardiomyocytes) has been identified as a molecular mediator of DOX cardiotoxicity.^22^ Top2 inhibition by DOX causes double-stranded breaks in DNA and activation of DNA damage response pathways involving p53-mediated apoptosis. Activation of p53 is followed by repression of genes involved in mitochondrial biogenesis and oxidative phosphorylation pathways. Mice with a cardiac-specific deletion of Top2β are protected from doxorubicin-induced cardiotoxicity.^22^

### Clinical Presentation

Cardiotoxicity may appear as asymptomatic or symptomatic reductions of left ventricular ejection fraction (LVEF) and may be acute or chronic.^23^ Anthracycline cardiotoxicity has several distinct presentations including acute, early-onset, and late-onset dependent on the time of exposure.^24^

Acute toxicity is uncommon (about 1%), and resembles an acute toxic myocarditis manifesting as transient left ventricular (LV) dysfunction, electrocardiographic changes, and arrhythmias, and develops immediately after a single dose, or a single course. Acute toxicity is believed to be reversible, and generally does not portend future development of heart failure.^3^,^24^–^26^ However, acute cardiac dysfunction may also reflect myocyte injury that eventually can evolve into early or late cardiotoxicity.^3^

Early-onset toxicity manifests within 1 year of anthracycline exposure^23^ and can lead to dilated cardiomyopathy. By definition, late-onset toxicity occurs more than 1 year after completion of therapy,^3^ presenting as progressive dilated cardiomyopathy. Its true incidence is not known, and it may represent post-anthracycline cardiac vulnerability to stressors and cardiovascular risk factors.^27^ The distinction between early- and late-onset is arbitrary and has recently been challenged. Cardinale et al. have shown that anthracycline cardiotoxicity occurs almost exclusively within the first year after completing treatment (98% of cases developed in the first year, with 3.5 months’ median time from the final dose of anthracycline to cardiotoxicity).^6^ These results suggest that late-onset anthracycline cardiotoxicity may reflect, at least in part, late diagnosis of early untreated cardiotoxicity.

### Risk Factors and Risk Assessment

Several treatment- and patient-related factors determine the risk for anthracycline cardiotoxicity (Table 1). Cumulative dose is the most important predictor of myocardial injury. Initial studies observed an exponential increase in the incidence of heart failure after a cumulative doxorubicin dose (5%, 26%, and 48% at cumulative doses of 400, 550, and 700 mg/m^2^, respectively).^23^ Subsequent studies suggested a lower threshold for cardiac dysfunction, with 7%, 9%, 18%, 38%, and 65% at cumulative doses of 150, 250, 350, 450, and 550 mg/m^2^, respectively.^32^ Other more recent studies support a cutoff of doxorubicin ≥250 mg/m^2^ or equivalent to define the high-exposure category.^28^,^33^,^34^ Table 2 depicts the maximal recommended dose for various anthracyclines.

**Table 1:** Risk Factors for Anthracycline Cardiotoxicity.^5^,^28^–^31^

Risk Factor
Compromised cardiac function (e.g. borderline low LVEF [50% to 55%], history of myocardial infarction, ≥moderate valvular heart disease) at any time before or during treatment
Older age (≥60 years) at cancer treatment
Female gender
Multiple cardiovascular risk factors (≥two risk factors)
Concomitant agents: trastuzumab, cyclophosphamide, paclitaxel
High-dose radiation therapy (≥30 Gy) where the heart is in the treatment field
Combination with alkylating or antimicrotubule chemotherapeutics
Young age (<5 years) at cancer treatment, especially girls Renal failure
Genetic factors (trisomy 21, hereditary hemochromatosis, African-American ancestry)

**Table 2:** Anthracycline Toxicity Equivalence Ratios.^35^

Anthracycline	Anthracycline Toxicity Equivalence Ratio	Maximal Recommended Cumulative Dose (mg/m2)
Doxorubicin	1	450
Daunorubicin	0.833†	600
Epirubicin	0.67	900
Idarubicin-IV	5	150
Mitoxantrone*	4	160
Liposomal anthracyclines		900

* Anthracenedione.

† Some studies reported a daunorubicin-to-doxorubicin cardiotoxicity equivalence ratio of 0.5.^35^,^36^

Patients are considered at high risk for anthracycline cardiotoxicity if receiving high-dose treatment (e.g. doxorubicin ≥250 mg/m^2^). Patients receiving lower-dose anthracycline (e.g. doxorubicin <250 mg/m^2^) are also considered at high risk if they have any of the risk factors in Table 1.^29^ From a clinical perspective, one may assume that any previous myocardial insult can potentially make the patient more susceptible to anthracycline-induced cardiotoxicity.^5^

Lower-dose anthracycline therapy (e.g. doxorubicin <250 mg/m^2^) with no other risk factors is usually well tolerated.^5^ However, a sizable individual variation exists in the susceptibility to anthracycline cardiotoxicity, and even low cumulative doses may modestly increase the risk for cardiac cardiotoxicity^29^ and may be associated with subclinical LV dysfunction.^37^

### Cardiac Imaging in Patients Undergoing Cancer Therapy

Routine surveillance imaging is recommended in asymptomatic patients considered to be at increased risk of developing cardiac dysfunction.^29^ Echocardiography is the cornerstone in the evaluation of patients prior, during, and after anthracycline therapy. However, echocardiography has low sensitivity for the detection of small reductions in LV function and reliably detects differences close to 10% in LVEF.^38^

Sub-clinical cardiotoxicity is commonly defined on cardiac imaging as a reduction in LVEF by >10% points to a value of EF <50% using echocardiography or equilibrium radionuclide angiography (MUGA).^6^,^39^,^40^ This definition has been endorsed by the American Society of Echocardiography and the European Association of Cardiovascular Imaging with a slight modification: LVEF decrease of >10% from baseline to a value <53%. This cutoff was chosen because data from six databases indicate that LVEF in the range of 53% to 73% should be classified as normal. The decrease in LVEF should be confirmed by repeated cardiac imaging, performed 2 to 3 weeks after the baseline diagnostic study showing the initial decrease in LVEF.^38^ This definition applies to all cancer therapeutics-related cardiac dysfunction (CTRCD).^38^

Global longitudinal strain (GLS) has been used to detect subtle alterations in systolic function, in an attempt to predict subsequent drops in LVEF.^39^,^41^ Reduction of GLS >15% from baseline occurring prior to any change in LVEF has been shown to precede the decrease in LVEF and can be used for the detection of subclinical LV dysfunction.^38^ The ANMCO/AIOM/AICO Consensus Document on clinical and management pathways of cardio-oncology accepts a GLS drop >10% as an indicator of subclinical LV dysfunction that warrants consideration of cardioprotection.^42^ However, there have been no studies to demonstrate that early intervention based on change in strain alone (in the absence of a reduction in LVEF) predicts the development of clinical heart failure or translates to a reduction in symptomatic cardiac dysfunction. The ongoing SUCCOUR trial^42^ may provide such information.

### Evidence to Inform Guidelines Regarding the Place of GLS for Surveillance for CTRCD

Three-dimensional echocardiography, when available, is the preferred technique for monitoring LVEF and detection of CTRCD. Advantages include better accuracy in detecting LVEF below the lower limit of normal and better reproducibility. Diastolic parameters have not yet demonstrated value in predicting subsequent CTRCD.^38^

Serial measurements of LVEF by MUGA are highly reproducible and have lower intra- and interobserver variability and a smaller coefficient of variability as compared with echocardiography, but have the disadvantage of radiation exposure.^38^

Cardiac magnetic resonance (CMR) is the reference standard in the evaluation of LV systolic function; CMR can be used when echocardiography is unreliable because of technical limitations, and when discontinuation of chemotherapy is being entertained.^29^,^38^

### Cardiac Biomarkers

Cardiac troponins are the sensitive biomarkers of myocardial injury and have been studied as indicators of early anthracycline-induced cardiotoxicity. The rationale for using cardiac troponins is that a reduction in LVEF becomes manifest only after a critical amount of myocardial damage has taken place, and it is therefore a relatively insensitive marker for cardiac toxicity.^43^ In a study of 703 patients with cancer, cardiac troponin I (TnI) was measured prior to chemotherapy, during the 3 days after the end of chemotherapy (early evaluation), and after 1 month (late evaluation).^44^ Evaluation of LVEF was carried out before chemotherapy, and 1, 3, 6, and 12 months after the end of the treatment, and again every 6 months afterward. Three TnI release patterns were identified, each associated with a different incidence of subsequent cardiac events. Patients with TnI consistently within the normal range showed no significant reduction in LVEF and had very low incidence of cardiac events (1%) during a 3-year follow-up. Patients with isolated TnI elevation at the early evaluation, and persistent TnI elevation at both early and late evaluations had high incidence of major adverse cardiac events, mainly asymptomatic LV dysfunction and heart failure (37% and 84%, respectively).^44^

Studies using modern ultrasensitive TnI assays confirmed that early increases in TnI are associated with subsequent cardiotoxicity in patients undergoing doxorubicin and trastuzumab therapy.^45^ Therefore, troponin can be used as a surrogate marker for subclinical myocardial toxicity. Importantly, the optimal timing of troponin measurements with regard to the specific chemotherapeutic regimen is not known.^43^ Some suggest baseline measurement followed by a single measurement with each cycle of chemotherapy, or after a cumulative dose ≥240 mg/m^2^ and before each additional 50 mg/m^2^.^46^ There is currently no data to indicate that troponin-based management of anthracycline-treated patients improves cardiac outcomes.

Although brain natriuretic peptide (BNP) and N-terminal pro-brain natriuretic peptide (NT-proBNP) are standard biomarkers used for the diagnosis of heart failure, their use as predictors of anthracycline cardiotoxicity remains to be established.^29^,^43^–^45^

### Cardiac Biopsy

The gold standard for detection of acute doxorubicin-induced cardiotoxicity is endomyocardial biopsy (EMB).^47^,^48^ Typical histopathological changes include vacuolization of the cytoplasm due to swelling of the sarcoplasmic reticulum and mitochondria and myofibrillar loss and/or myofibrillar disarray.^49^,^50^ Electron microscopy demonstrates loss of myofibrils and distention of the sarcoplasmic reticulum and T-tubules. Endomyocardial biopsy can be used to grade the severity of cardiotoxicity using electron microscopy based on the percent of cells demonstrating typical changes (seven-point scale described by Billingham et al.).^48^,^51^ Moreover, EMB demonstrates morphologic changes with low cumulative dose (e.g. 200 mg/m^2^).^52^ However, the correlation of biopsy scores with non-invasively assessed LVEF is poor,^52^ representing the ability of the left ventricle to compensate.^5^

Yet EMB is rarely used, owing to the invasive nature of the procedure and the availability of simpler sensitive markers of cardiotoxicity (e.g. troponin, longitudinal strain) and because histological evidence for cardiotoxicity can exist without evidence of cardiac dysfunction, which may not contribute to clinical decisions.

### Clinical Follow-up

Identifying cardiotoxicity at a preclinical stage is paramount because an asymptomatic decrease in LVEF generally precedes clinically overt heart failure,^53^ and because the potential for LV recovery increases with initiation of therapy at the preclinical stage.^6^,^7^

Schwartz et al. were the first to show that monitoring LVEF with serial measurements of radionuclide angiocardiography can reduce the incidence of doxorubicin-induced heart failure.^54^ Discontinuation of doxorubicin therapy was recommended for a decrease in LVEF of ≥10% and/or to a final LVEF of ≤50% in patients with normal baseline LVEF (≥50%), and for a decrease of ≥10% and/or a final LVEF 30% in patients with baseline LVEF of 30%–50%. Doxorubicin therapy was not initiated with baseline LVEF <30%. The incidence of clinical heart failure was 2.9% versus 20.8% in patients managed with and without these criteria, respectively.^54^

While the importance of early identification of asymptomatic LV dysfunction is undisputed,^29^ there are no studies that compare the efficacy of different cardiac surveillance protocols in cancer survivors. The follow-up protocol with regard to the timing and frequency of surveillance is based on clinical judgment and patient circumstances and varies by institution. For example, the Stanford Cardiology recommendations for asymptomatic cardiac monitoring of anthracycline-treated patients include echocardiography at baseline LVEF assessment, 200 mg/m^2^ (or at end of 240 mg/m^2^ if planned total dose), 300 mg/m^2^, 400 mg/m^2^, and every 50 mg/m^2^ thereafter (doxorubicin equivalents).^55^

Another proposed schedule includes measurement of LVEF at 6 months following treatment, annually for 2 to 3 years thereafter, and then at 3- to 5-year intervals for life, with more frequent monitoring in high-risk patients.^30^ This protocol may not be appropriate in patients with low LVEF (30%–50%).^54^

The American Society of Clinical Oncology Clinical Practice Guideline recommends a baseline echocardiogram, followed by an echocardiogram between 6 and 12 months after completion of cancer-directed therapy in asymptomatic patients considered to be at increased risk of cardiac dysfunction (Table 1).^29^ The frequency and duration of surveillance in patients at increased risk who are asymptomatic and have no evidence of cardiac dysfunction on their 6- to 12-month post-treatment echocardiogram are not clear,^6^ but reassessment of cardiac function is reasonable 3–5 and 10 years after anthracycline therapy.^30^,^31^

**Figure 1:**
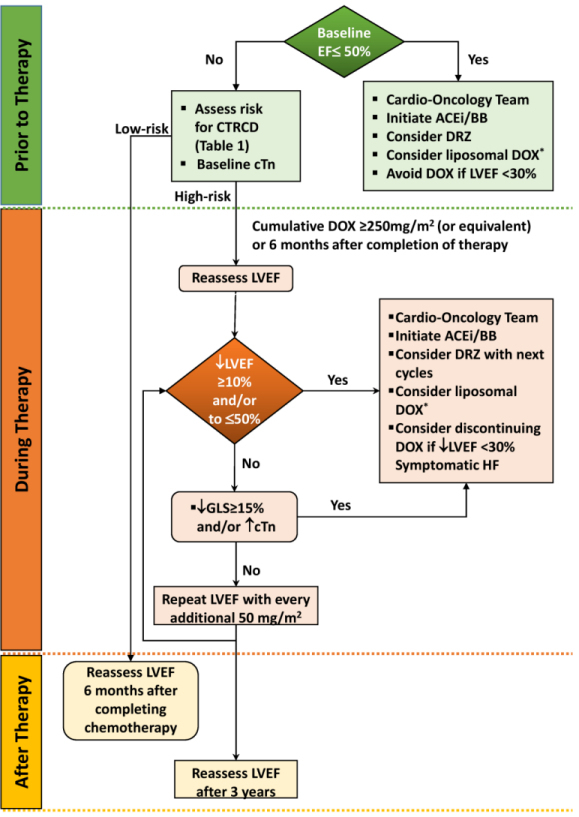
Proposed Protocol for Early Detection of Subclinical LV Dysfunction and Management of Cardiotoxicity in Patients Receiving Anthracyclines. ^*^Approved by the FDA in acquired immune deficiency syndrome (AIDS)–related Kaposi sarcoma, advanced/refractory ovarian cancer, multiple myeloma after failure of at least 1 prior therapy, and metastatic breast cancer.^57^,^58^ ACEi/BB, angiotensin-converting enzyme inhibitor/β-blocker; cTn, cardiac troponin; CTRCD, cancer therapeutics–related cardiac dysfunction; D/C, Discontinue; DOX, doxorubicin; DRZ, dexrazoxane; EF, ejection fraction; GLS, global longitudinal strain; HF, heart failure; LVEF, left ventricular ejection fraction.

It is recommended to discontinue doxorubicin (at least temporarily) in patients who develop clinically overt heart failure.^56^ The indications for withdrawal or withholding of therapy in patients with asymptomatic LVEF decline are less clear, although this strategy reduces the incidence of clinical heart failure.^54^ A LVEF reduction of >20% from baseline (even in the presence of normal LVEF) requires reconsideration of therapy and further frequent clinical and echocardiographic examinations (Figure 1).^31^ With smaller reductions in LVEF, angiotensin-converting enzyme inhibitor (ACEi) and β-blocker therapy should be initiated, with withdrawal of therapy and reevaluation after 1 month.^59^

Of note, protocols for screening and monitoring patients for anthracycline cardiotoxicity ignore potential oncologic benefits, which vary among different tumor types, stage of the disease, and overall prognosis. There are no specific recommendations regarding continuation or discontinuation of cancer therapy in these patients. This decision, made by the oncologist, should be informed by close collaboration with a cardiologist, fully evaluating the clinical circumstances and considering the risks and benefits of continuation of therapy.^29^

### Prevention Strategies for Anthracycline Cardiotoxicity

#### Dexrazoxane

Dexrazoxane (DRZ; Cardioxane®, Clinigen Healthcare Ltd, Burton on Trent, United Kingdom) is a cardioprotective agent for anthracycline-induced cardiotoxicity.^57^,^60^–^64^ Dexrazoxane is a *bis*-ketopiperazine which undergoes hydrolysis of its piperazine rings and releases a diacid diamide (an analogue of ethylene diamine-tetraacetic acid [EDTA]). Diacid diamide chelates redox-active iron before it converts O_2_^−^ and H_2_O_2_ into more potent hydroxyl radicals or equally reactive iron–oxygen complexes. Dexrazoxane decreases mitochondrial iron levels, thereby preventing the formation of anthracycline-iron complexes and subsequent ROS formation.^15^,^20^,^48^ Another major mechanism of the protective action of DRZ is related to its ability to compete on ATP-binding sites on Top2β, producing a configuration change which prevents complex formation with anthracycline.^22^,^65^

The efficacy of dexrazoxane has been demonstrated in two randomized trials in breast cancer patients^63^,^64^ and in children with acute lymphoblastic leukemia.^64^,^66^ In a Cochrane meta-analysis, use of DRZ was associated with a marked reduction in the risk for clinical heart failure after anthracycline therapy (relative risk 0.18, 95% CI 0.1–0.32, *P*< 0.001).^61^ Dexrazoxane also attenuates the increase in troponin levels after anthracycline use.^64^

Because DRZ concomitantly inhibits formation of drug-induced Top2α–DNA cleavage complexes, there is a concern that DRZ may render anthracyclines less effective against cancer cells. However, a meta-analysis of randomized clinical trials found no evidence that dexrazoxane lowers doxorubicin’s anticancer effects.^61^

Another concern is the potential risk of increased secondary malignancy. Two randomized open studies reported a 3-fold increase in the incidence of second hematologic malignancies, particularly acute myeloid leukemia and myelodysplastic syndrome in DRZ-treated children.^64^,^66^ An increased risk of severe myelosuppression and severe infection was also reported.^67^

Based on these reports, the European Medicines Agency initially restricted the use of DRZ to adults with advanced or metastatic breast cancer who have received a cumulative dose of at least 300 mg/m^2^ doxorubicin or 540 mg/m^2^ epirubicin before starting DRZ.^68^ In the US, the Food and Drug Administration (FDA) has also approved DRZ only in patients who have received more than 300 mg/m^2^ of doxorubicin for metastatic breast cancer and who may benefit from continued doxorubicin treatment.^69^ However, long-term follow-up (5 to 10 years) of childhood acute lymphocytic leukemia patients found no differences in the incidence of secondary malignancies between dexrazoxane and placebo,^70^,^71^ suggesting that more widespread use of DRZ may be appropriate. More recently, the European Medicines Agency narrowed the contraindications for dexrazoxane to include only patients under 18 years old who are intended to receive a total cumulative dose of doxorubicin <300 mg/m^2^.^72^ The American Society of Clinical Oncology Clinical Practice Guideline also supports the use of DRZ in patients planned to receive high-dose anthracyclines (e.g. doxorubicin ≥250 mg/m^2^, epirubicin ≥600 mg/m^2^).^29^

Dexrazoxane is administered as a short intravenous infusion immediately prior to a bolus dose of doxorubicin at a recommended dose of 10 mg per 1 mg of doxorubicin or epirubicin.^73^ Because myocardial uptake of DRZ is very rapid and approaches its maximum level within 1 min, infusion just prior to the anthracycline administration is appropriate. Doxorubicin is given within 30 minutes after the completion of dexrazoxane administration. Reducing the dosage by 50% is indicated in patients with moderate to severe renal impairment (creatinine clearance values less than 40 mL/min), i.e. dexrazoxane-to-doxorubicin ratio reduced to 5:1.

#### Liposome-encapsulated anthracyclines

Liposomal encapsulation of anthracyclines modifies pharmacokinetics and tissue distribution without compromising tumoricidal efficacy.^57^ Liposomal doxorubicin is restricted to the intravascular space when capillary structure is intact with tight junctions, such as in the heart, and liposome accumulation in the myocardium is reduced. By contrast, liposomal formulations are small enough (80–90 nm) to penetrate through the more fragile fenestrated microvasculature that characterizes solid tumors, resulting in preferential accumulation in tumors and minimal release in plasma and healthy tissues, voiding the high plasma levels of free doxorubicin, which is strongly associated with cardiac toxicity.^57^,^74^,^75^

Two liposomal formulations have been approved for clinical use. Pegylated (polyethylene glycol embedded in the lipid layer) liposomal doxorubicin (PLD), also known generically as lipodox (Doxil®, Caelyx®), was the first FDA-approved cancer nanomedicine.^76^ Pegylation provides a highly hydrophilic, protective cover to liposomes, avoiding their detection by the mononuclear phagocyte system and thereby prolonging blood circulation time.^74^ The uncoated formulations of doxorubicin (Myocet®, a liposome-encapsulated doxorubicin citrate complex; or DaunoXome®) include citrate-increasing doxorubicin encapsulation.^75^,^77^

In the treatment of metastatic breast cancer, uncoated liposomal doxorubicin achieved a response comparable to that of conventional doxorubicin. However, the median cumulative anthracycline dose at the onset of cardiotoxicity was 785 mg/m^2^ and 2,220 mg/m^2^ for the liposomal formulation versus 570 mg/m^2^ and 480 mg/m^2^ mg/m for free drug in the studies of Harris et al.^78^ and Batist et al.,^79^ respectively. In first-line therapy for metastatic breast cancer, pegylated liposomal doxorubicin provided comparable efficacy to doxorubicin, with significantly reduced cardiotoxicity.^80^

When EMB was used to detect anthracycline-induced cardiac damage, PLD-treated patients had significantly lower biopsy scores compared with those of doxorubicin controls despite higher cumulative doses of anthracycline.^81^ A Cochrane analysis identified liposomal anthracyclines as the only formulations able to reduce the risk of anthracycline-related cardiotoxicity.^82^

The efficacy and cardiac safety of liposomal doxorubicin has also been documented under conditions of increased risk for cardiotoxicity such as concomitant administration of trastuzumab in breast cancer treatment.^83^–^85^ Because of cost considerations, liposomal doxorubicin is approved by the FDA in acquired immune deficiency syndrome (AIDS)-related Kaposi sarcoma, advanced/refractory ovarian cancer, multiple myeloma after failure of at least one prior therapy, and metastatic breast cancer.^57^,^58^

#### Prolonged administration

Anthracycline myocardial concentrations are higher after a bolus dose, and therefore cardiotoxicity is related to peak levels (C_max_).^57^,^75^ By contrast, anthracycline activity correlates with total exposure to anthracyclines, or the area under the curve (AUC). Therefore, administering anthracyclines via continuous infusion rather than as a bolus dose has been proposed to limit peak dose levels and reduce anthracycline-related cardiac effects. This approach minimally affects anthracycline AUC, and diminishes C_max_ and anthracycline myocardial accumulation.^75^ Increasing infusion duration (>6 hours) reduced cardiotoxicity without compromising the therapeutic efficacy,^57^,^86^,^87^ albeit not in all studies.^88^ Biopsy data also support cardiac protection for 72-h and 96-h infusions.^89^

Clinicians may choose to use either dexrazoxane, continuous infusion (range, 6 to 96 hours),^10^ or liposomal formulation of doxorubicin,^10^,^84^ for prevention of cardiotoxicity in patients planned to receive high-dose anthracyclines (e.g. doxorubicin ≥250 mg/m^2^).^3^,^29^ Clinical experience suggests that these therapies are underused even in high-risk patients.^89^

### Medical Therapy

Patients developing reduced LVEF during or following anthracycline treatment should be treated according to current guidelines. In patients with asymptomatic LVEF reduction, current guidelines recommend initiation of ACEi and β-blockers.^3^,^29^,^90^ The expectation is that treatment with these agents will reduce neurohormonal activation, reduce adverse LV remodeling, and prevent or delay the onset of symptoms.^90^ These therapies appear to be beneficial in patients with anthracycline cardiotoxicity, especially when initiated early.^6^,^7^

Depending on the perceived benefits from the continuation of therapy, it is sometimes possible to continue treatment with the support of ACEi and β-blockade.^59^ Liposomal anthracycline or DRZ may also be considered in this setting.^3^,^65^

Prophylactic use of ACEi and β-blockers, prior to any documented reduction in LVEF, has been studied in all anthracycline-treated patients^91^ and in high-risk patients such as those planned for high-dose anthracyclines,^92^ those with elevated troponin,^93^ or concomitant trastuzumab therapy.^94^ Although some studies demonstrated benefit,^92^ this strategy remains inconclusive as some studies were neutral^94^–^96^ or demonstrated modest benefits.^91^,^95^,^97^ In patients with evidence of early cardiac toxicity manifesting as cardiac troponin elevation or GLS reduction without a reduction in LVEF, initiation of medical therapies may be a sensible option.^3^,^59^,^69^,^93^

## PROTEASOME INHIBITORS

Proteasome inhibitors (PIs) are used in multiple myeloma, Waldenstrom macroglobulinemia, low-grade non-Hodgkin lymphomas, and primary amyloidosis. In these patients, clinical trials with PIs involved patients with advanced disease who had been previously treated with potentially cardiotoxic regimens and might suffer from amyloid deposition or hyperviscosity. The extent to which the cardiac events reported with PIs are due to patients’ baseline comorbidities, toxicity from prior treatments, cardiac involvement of multiple myeloma, carfilzomib itself, or a combination of these factors, can be difficult to determine.

### Bortezomib (BTZ)

Bortezomib (Velcade®; Millenium Pharmaceuticals, Cambridge, MA, USA) is a first-in-class PI, acting as a reversible inhibitor. San Miguel and colleagues studied grade ≥3 heart failure event rates in 2,509 patients treated with BTZ in phase II and III trials and an additional 1,445 patients treated with non-BTZ-based therapies in the control arms of the phase III studies.^98^ The rate of heart failure events was approximately 2.0% in both the BTZ and control groups.

In a meta-analysis that included 11 phase III and 14 phase II trials of patients who received bortezomib (*n*=4,330), high-grade cardiovascular toxicity including heart failure and sudden cardiac death occurred in 2.3% of patients, but bortezomib did not significantly increase the risk of all-grade and high-grade cardiotoxicity.^99^

Overall, although reports of bortezomib-induced heart failure and LV systolic dysfunction exist, over 10 years of large-scale use of this agent indicates that the incidence of heart failure is apparently very low.^100^

### Carfilzomib (CFZ)

Carfilzomib (Kyprolis®; Amgen Inc., Thousand Oaks, CA, USA) is more potent than bortezomib and irreversibly binds to the active sites of the 20S proteasome; it is used in patients with relapsed/ refractory myeloma. In an integrated safety analysis of four phase II trials in which carfilzomib monotherapy was used in multiple myeloma patients, 7.2% had heart failure events (including pulmonary edema and decreased ejection fraction).^101^

Among 60 consecutive myeloma patients treated with carfilzomib-based regimens, 12% experienced a relative reduction of LVEF of ≥20% (median of 6 months from initiation of therapy).^102^ There was a time-dependent increase in the incidence of LVEF reduction: 5% at 3 months, 8% at 6 months, 10% at 12 months, and 12% at 15 months. Levels of NT-proBNP increased concomitantly with LVEF reduction in all patients, without an increase in troponin levels. After temporary discontinuation of CFZ, the LVEF returned to baseline in all patients after a median of 60 days. Similar reversibility of the cardiotoxic effect of CFZ has been reported in other studies.^101^,^103^,^104^ Overall, cardiotoxicity is higher with CFZ as compared with bortezomib.^104^

### Ixazomib

Ixazomib (Ninlaro®; Millennium Pharmaceuticals, Cambridge, MA, USA) is the first oral PI and has recently received a license for relapsed and refractory multiple myeloma. Ixazomib (MLN9708) is a novel oral proteasome inhibitor used in the treatment of AL-amyloidosis and multiple myeloma. Although cardiac toxicity manifesting as reduction in LVEF and clinical heart failure has been reported,^106^ the patients were exposed to multiple chemotherapeutic regimens with varying degrees of cardiotoxicity.

## HUMAN EPIDERMAL GROWTH FACTOR RECEPTOR 2 (HER2)-TARGETED THERAPIES

### Oncological Therapeutic Target

The human epidermal growth factor receptor 2 (HER2) signaling pathway has a key role in several malignancies (predominantly in breast cancers, for which around a quarter of the cells overexpress HER2), and its blockade has been shown to significantly halt cancer progression.^107^ Trastuzumab (Herceptin®; Genentech, San Francisco, CA, USA), the most dominant member of the HER2-targeted therapy, is a monoclonal antibody that confers the ability to reduce the proliferative and metastatic potential of HER2-overexpressing cancer cells. Trastuzumab therapy in combination with additional chemotherapeutic agents (including anthracycline) is associated with significant improvement in overall and disease-free survival both for early-stage^108^,^109^ and late-stage^110^ breast cancer patients.

### Cardiotoxicity Mechanism

The tyrosine kinase transmembrane receptor HER2 is encoded by the ERBB2 gene in human.^111^ It is a key regulator of cardiomyocyte growth and proliferation and an active player in cardiomyocyte response to stressogenic stimuli as a pro-survival pathway.^112^ Trastuzumab binds to subdomain IV of the extracellular domain of HER2 and inhibits HER2 signaling. Pertuzumab binds to subdomain II of the tyrosine kinase receptor, and following its binding it prevents heterodimerization with HER3 and thereby prevents tyrosine kinase activation in a different mechanism, which may translate into synergistic clinical efficacy.^113^ Ado-trastuzumab emtansine is composed of a cytotoxic agent delivered specifically to HER2-expressing cells and thus may carry a lower risk for cardiotoxicity.^114^

The HER2 ligand neuregulin-1 is primarily released from endothelial cells and activates the ERBB2 signaling pathway in cardiomyocytes, which includes activation of phosphoinositide 3-kinase, protein kinase A, and the mitogen-activated protein kinase pathway. The ERBB2 signaling pathway is highly connected with the cellular response to oxygenic stress. For example, neuregulin-1 activation results in activation of heat shock proteins, a well-known family of protein stabilizers, significantly upregulated following exposure to ROS.^115^–^117^ Therefore, it is not surprising that ERBB2 may also be activated by anthracycline toxicity.^118^ This led to the hypothesis that anthracycline-trastuzumab therapy may serve as a “two-hit” phenomenon, the first “hit” producing oxidative stress and DNA damage and the second compromising the pro-survival cardioprotective mechanisms.

### Cardiotoxic Impact

The HER2-targeted therapies have been associated with the development of LV systolic dysfunction and the development of clinical heart failure. Trastuzumab therapy was shown to augment and presumably synergize the cardiac toxicity of anthracyclines when combined with those agents. In the first phase III clinical study evaluating trastuzumab in breast cancer patients,^107^ 16% of the patients receiving an anthracycline–trastuzumab combination developed severe heart failure (NYHA III–IV) versus 3% in the anthracyclines group. Subsequent randomized clinical studies observed lower rates of heart failure development (e.g. only 0.8% for severe heart failure in the HERA trial), but this may be due to higher awareness as well as strict and focused protocols that did not recruit high-risk cardiovascular patients and prevented concurrent anthracycline administration (Table 3).^108^,^119^,^121^ Moreover, a recent long-term follow-up (approximately 100 months post treatment) study showed that HER2-targeted therapy did not serve as a significant risk factor for clinical heart failure development, implying a limited potential early toxicity or reversibility of the cardiotoxic effect.^111^

**Table 3:** Selected Major Clinical Studies Involving HER2-targeted Therapies.

Clinical Study	Trastuzumab-Anthracycline Interval	Monitoring	Cardiac Risk-related Exclusion Criteria	Clinical Heart Failure (%)	LV Dysfunction (%)	Partial Reversibility Reported
Slamon et al.^107^	Concurrent	None pre-specified	None	16%	27%	Yes
Romond et al.^109^	3 weeks	MUGA	LVD, HTN, ANY	4.1%	19%*	Yes
Polman et al.^118^	3 weeks	MUGA or ECHO	LVD, HTN, ANY	2%	19%	Yes
Goldhirsch et al.^119^	3 months	MUGA or ECHO	LVD, HTN, ANY	0.8%	7.2%	Yes
Tolaney et al.^120^	No anthracycline given	MUGA or ECHO	LVD, HTN, ANY	0.5%	3.2%	Yes

* Discontinued drug due to cardiac adverse event.

ANY, any sign or symptom of cardiac disease; ECHO, echocardiography; HTN, hypertension; LVD, left ventricular dysfunction.

Overall, trastuzumab therapy confers a significant risk for heart failure development, with a relative risk of 5.1 based on a Cochrane meta-analysis.^122^ Trastuzumab cardiotoxicity is more subtle when anthracycline-free regimens are used,^122^ and there is conflicting evidence regarding its reversibility and reoccurrence in the case of re-exposure to the drug.^123^

Therapy using HER2 in combination with pertuzumab and trastuzumab is more efficacious than HER2 alone because of more complete signaling blockade.^125^ Using the standard definition,^38^ similar cardiotoxicity rates were observed with the addition of pertuzumab to trastuzumab and docetaxel, and reversibility occurred in the majority of patients.^125^ In another study, cardiotoxicity was numerically higher with pertuzumab, but overall lower than 1%.^126^ A relatively safe cardiac risk profile was also demonstrated for other HER2-directed therapies such as lapatinib and trastuzumab emtansine.^126^,^127^

Collectively, HER2-targeted therapies confer a significant risk for cardiotoxicity; however, the majority of evidence indicates that this risk is mostly related to concurrent or preceding anthracycline therapy. Hence, anthracycline-free regimens should be considered for patients with HER2-positive breast cancer, especially when these patients have high risk factors.

### Risk Factors

The main risk factor for HER2-targeted therapy cardiac toxicity is past administration of anthracyclines.^128^ Cumulative anthracycline dosage is a significant modifier of the risk incurred by anthracyclines, with dosages >250 mg/m^2^ of doxorubicin and >600 mg/m^2^ of epirubicin^119^,^120^ appearing to carry a higher risk (i.e. a dose considered high-risk without concomitant HER2-targeted therapy).^29^ Beyond dosages, the length of time between anthracycline and HER2-targeted therapies is inversely correlated with the risk, with concurrent administration conferring the highest risk. While trastuzumab dosage has not been found to be a risk factor, the cumulative dosage of trastuzumab and the length of therapy was found to be a significant modifier of the relative risk in the recent publication of 11 years of follow-up from the HERA trial.^129^

**Figure 2:**
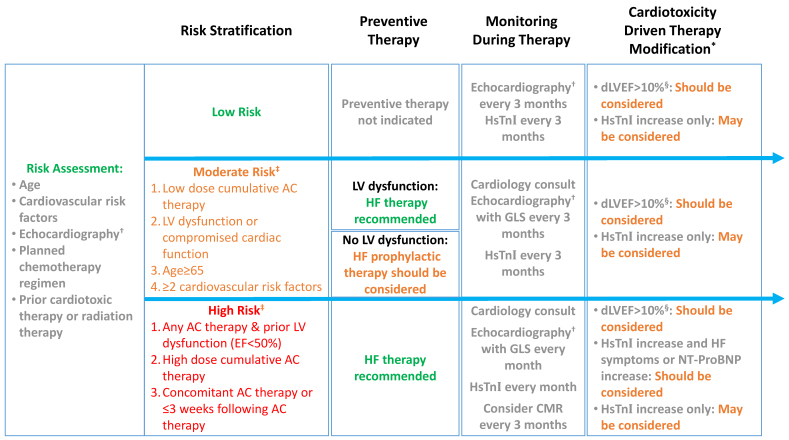
Risk Stratification, Preventive Therapy, and Surveillance of Trastuzumab-treated Patients. Blue arrows symbolize cardio-oncological chronological point of intervention. *Dependent on the availability of alternative therapies and following cardio-oncology team discussion. Holding trastuzumab and re-challenge following EF normalization (LVEF>50%) is one potential strategy. ^†^LVEF should be assessed using 2D Simpson’s LVEF or preferably 3D-based LVEF. ^‡^One risk stratification criterion required. ^§^dLVEF>10%: reduction in LVEF>10% and LVEF<50%. AC, anthracyclines; dLVEF, difference in left ventricular ejection fraction; EF, ejection fraction; GLS, global longitudinal strain; HsTnI, high-sensitivity troponin I; LV, left ventricular; RT, radiation therapy.

Age is a significant risk modifier for trastuzumab cardiotoxicity. Older patients (with a risk cut-off age of 60 to 70) tend to have a higher risk for developing cardiotoxicity according to most studies.^29^,^109^,^110^,^130^ Patients with low baseline LVEF are at high risk. This patient population was excluded from most randomized studies, but according to real-world data low baseline LVEF is a major risk factor.^131^ Additional risk factors that should be considered for stratifying patients include: obesity (BMI≥30), coronary artery disease, hypertension, LV valvular disease, smoking, and renal failure.^29^,^130^ We do not have evidence for effective preventive therapy for trastuzumab cardiotoxicity. There are contradicting results regarding the effectiveness of β-blockers and ACEi for preventing cardiotoxicity, especially for those patients with no other indications for these therapies.^94^,^132^ A recent randomized controlled trial by Guglin et al. (presented at the American College of Cardiology Conference 2018) demonstrated no beneficial role for either β-blocker therapy or ACEi for patients undergoing trastuzumab therapy.^133^ Figure 2 depicts the risk stratification, preventive therapy, and surveillance of trastuzumab-treated patients based on the European Society of Cardiology guidelines and the American Society of Clinical Oncology guidelines.^3^,^29^

## VASCULAR ENDOTHELIAL GROWTH FACTOR INHIBITORS

### Oncological Therapeutic Target

Inhibitors of vascular endothelial growth factor (VEGF) signaling include both monoclonal antibodies and small molecules. Bevacizumab (Avastin®; Genentech, San Francisco, CA, USA), a monoclonal antibody, binds to the VEGF receptor and inhibits its downstream signaling pathway. Bevacizumab has been found to improve survival in multiple solid tumors.^134^,^135^ Small molecules affecting VEGF signaling are mostly non-specific tyrosine kinases, some of which are associated with LV dysfunction. The prototype drugs that pertain to this group include: sorafenib, approved for renal cell carcinoma and hepatocellular carcinoma as well as resistant thyroid carcinoma; and sunitinib, approved for gastrointestinal stromal tumor and renal cell carcinoma.^136^,^137^

### Cardiotoxicity Mechanism

Hypertension, arterial thromboembolism, pulmonary hypertension, cardiac ischemia, and QT prolongation are the main adverse effects associated with VEGF-inhibiting agents.^138^,^139^ Cardiomyopathy is a relatively infrequent adverse event of VEGF inhibitors in the absence of additional insults and may predominantly occur when other cardiovascular adverse event ensue (i.e. severe hypertension). Vascular endothelial growth factor has a pivotal role in cardiovascular homeostasis and is responsible for microvascular plasticity required during stressogenic events such as ischemia or pressure overload. Inhibition of VEGF signaling and the closely related tyrosine kinase, p38 mitogen-activated protein kinase (MAPK), may result in maladaptive response, hastening the transition to heart failure when pressure overload is induced.^140^ In addition, small molecules designed to inhibit VEGF are relatively non-selective, a property augmenting their cardiotoxic profile. These inhibitors have been shown to be involved in inhibiting several tyrosine kinases such as the PDGF pathway (known as key pro-survival factor for cardiomyocytes), the RAF1-related ERK signaling (known to be involved in the balance between eccentric and concentric cardiac growth),^141^ as well as the 5’ AMP activated protein kinase (involved in metabolic adaptation to energetic stress).^142^

### Cardiotoxic Impact

The most common cardiovascular adverse event of this group of drugs is hypertension, occurring in 25%–70% depending on the specific drug and dosage. Cardiomyopathy is a dreadful but relatively less common adverse event.^138^ The risk for cardiomyopathy is more drug-dependent than a class effect for this group of drugs. Bevacizumab therapy results in heart failure in less than 5% of the patients.^143^ Risk factors include prior cardiotoxic therapy and baseline LV dysfunction.^144^ Small molecular tyrosine kinase inhibitors (TKIs) are associated with higher risk for cardiomyopathy, with up to 15% of the patients developing LV dysfunction and up to 10% developing clinical heart failure. A meta-analysis of all VEGF-TKI agents tempered the attributable risk and identified the risk for heart failure as 3.2% for the group, with a significant odds ratio of 2.4.^145^ One should remember that the real risk of these drugs is underestimated since they are mainly given for patients with metastatic disease, and therefore longitudinal long-term surveillance of their detrimental effects is impossible.

There is a lack of sufficient data regarding the risk of these agents, and specific guidelines^29^ mandate careful surveillance protocol. We suggest using a similar protocol to that used for trastuzumab (Figure 2), with specific attention to identifying hypertension (which may worsen heart failure) and QT prolongation.

## IMMUNE CHECKPOINT INHIBITORS

### Oncological Therapeutic Target

The immune checkpoint inhibitors (ICPi) were designed in order to release specific constraints from the host immune system, enabling more potent response toward cancer cells. Tumor cells exploit immune regulatory mechanisms to evade the immune system by activating two negative regulatory mechanisms for T cell response (proliferation, pro-survival, cytotoxicity): the programmed death 1 (PD1)/PD1 ligand (PD-L) and the cytotoxic T-lymphocyte associated antigen 4 (CTLA4)/B7.^146^

The immune checkpoint inhibitor class of drugs aims at reversing the immune system inhibition induced by cancer cells. This class includes inhibitors of CTLA4 such as ipilimumab, programmed death 1 inhibitors such as pembrolizumab and nivolumab, and the PD1 ligand inhibitor atezolizumab. The main therapeutic indications include melanoma, non-small cell lung cancer, renal cell carcinoma, and Hodgkin’s disease.

### Cardiotoxicity Mechanism

The immune checkpoint inhibitors are usually well tolerated but, as may be expected, confer a significant risk for immune-system related adverse events that may limit the therapy or require discontinuation in up to 40% of patients. Based on their mechanism of action, these agents destabilize the balance between self-tolerance and autoimmunity, and induce immune-system related adverse events such as hepatitis, pneumonitis, colitis, dermatitis, and myositis.^147^ The anti-CTLA4 therapy targets the immune system inhibition in a more robust way and also relatively upstream within the immune activation cascade, whereas the anti-PD1 therapies act at a later stage mostly relevant to peripheral tissues. This mechanistic variance has two important consequences. First, the adverse event profile is different for the anti-CTLA4 and the anti-PD1 therapies. The anti-CTLA4 therapies are associated with more severe and systemic autoimmune adverse events such as colitis and hypophysitis; the anti-PD1 therapies are associated with less severe and somewhat peripheral reactions such as pneumonitis and thyroiditis.^148^ Second, given the fact that these agents act on different steps of the immune cascade, they might have a positive but also negative synergetic effect when combined.^147^

Reports about the cardiotoxicity of the immune checkpoint inhibitors are relatively scarce; however, the toxicity is severe and significant, and it may result in fatal events. The incidence of this hazardous event is generally low, and the main risk factor is the combination of anti-CTLA4 and anti-PD1 agents.^147^,^149^,^150^ Johnson et al. reported about two cases of fulminant myocarditis resulting in fatality due to combined therapy with ipilimumab (anti-CTLA4) and nivolumab (anti-PD1). Interestingly, they further characterized the infiltrating lymphocyte inducing the myocarditis by performing next-generation sequencing for the T cell receptors. Based on this evaluation, they identified T cell populations with predominant cardiac infiltration and clonal expansion, which suggested a potential role for antigen resemblance between the tumor, skeletal muscle, and cardiac tissue. Further supporting this finding, the authors report that within the tumors of these patients they identified T cells targeting muscle-specific antigens such as troponin and desmin.^149^

### Cardiotoxic Impact

The incidence of myocarditis following immune checkpoint inhibitors therapy is undoubtedly underestimated since patients in clinical trial were not monitored for this adverse event (troponin surveillance and echocardiography were not routinely conducted).^147^ Among 20,594 patients from the safety database of Bristol–Myers Squibb corporate, 18 cases (0.09%) of severe myocarditis were reported for patients receiving immune checkpoint inhibitors. The risk for combined therapy was significantly higher (0.27% combined versus 0.09% monotherapy with PD1 inhibitor).^149^ Recent data from a multicenter registry indicate that the prevalence of myocarditis is higher. Myocarditis rates based on a single center (Massachusetts General Hospital) are 1.14% for all immune checkpoint inhibitors, 0.5% of patients on anti-PD1 alone, and 2.4% with combined anti-PD1/anti-CTLA4 antibodies.^150^

Myocarditis occurs early during treatment and was more common with combination immune checkpoint inhibitor (ICPi) therapy.^150^ The clinical features include a concomitant additional autoimmune adverse reaction in most cases (usually a rash, hepatitis, or myositis). Development of a progressive heart failure that persists following discontinuation of the immune checkpoint therapy is characteristic. Atrial and ventricular arrhythmias and conduction abnormalities have been reported in several patients.^147^,^149^,^151^ The imaging and laboratory findings are similar to those of myocarditis from other etiologies including the cardiac MR findings, troponin and BNP levels, and histopathological immunostaining. Serum troponin is elevated in the majority of patients (>90%), whereas the LVEF and natriuretic peptides may be normal.^150^ Therefore, obtaining troponin levels at baseline and with each cycle may be reasonable, and an increasing troponin level should warrant consideration of myocarditis.^150^

**Figure 3:**
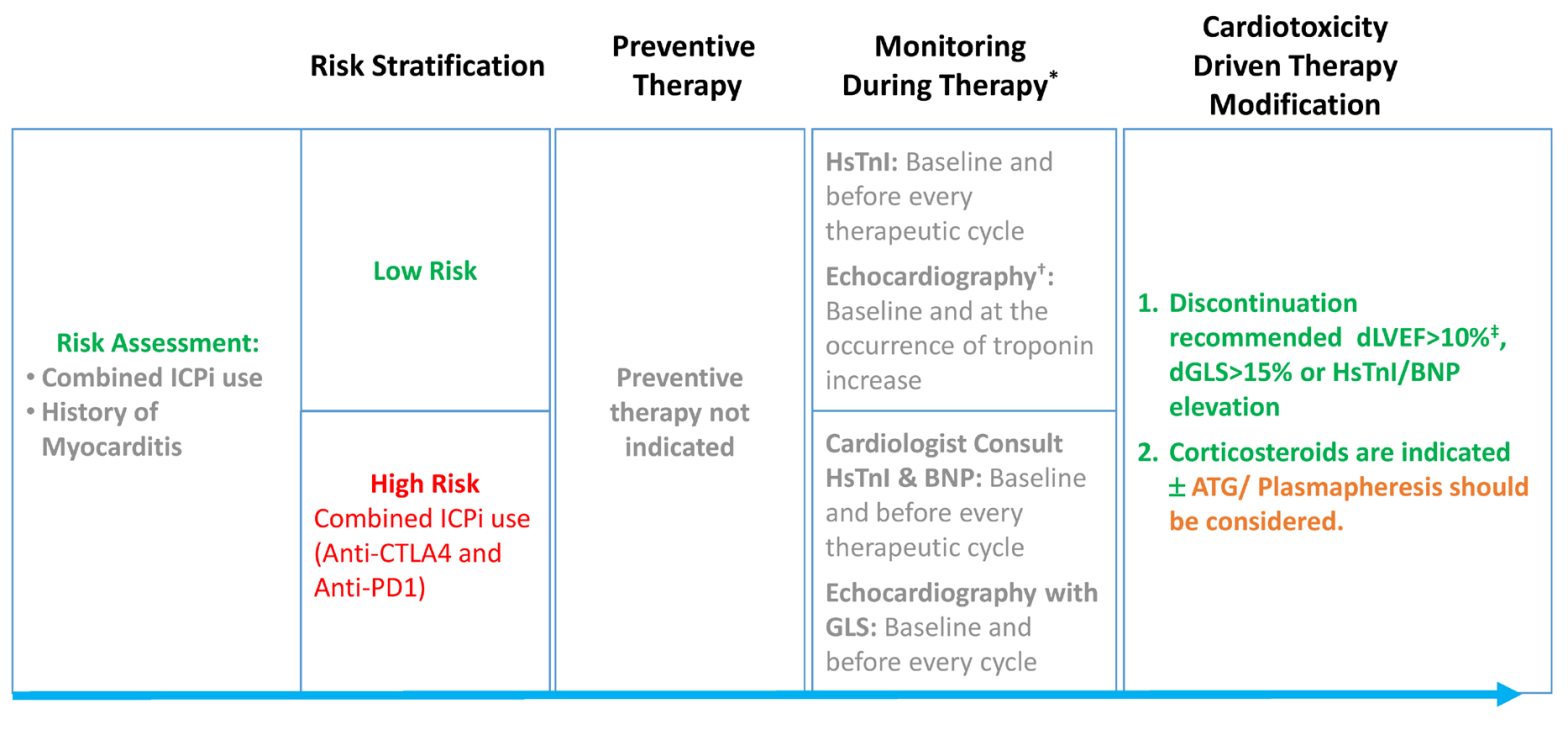
Risk Stratification, Preventive Therapy, and Surveillance of Immune Checkpoint Inhibitor-treated Patients. Blue arrow symbolizes cardio-oncological chronological point of intervention. *Dependent on the availability of alternative therapies and following oncologist consultation; †LVEF should be assessed using 2D Simpson’s LVEF or preferably 3D-based LVEF; ‡dLVEF>10% to LVEF<50%. ATG, antithymocyte globulin; dLVEF, difference in left ventricular ejection fraction; dGLS, difference in global longitudinal strain; EF, ejection fraction; GLS, global longitudinal strain; HsTnI, high-sensitivity troponin I; ICPi, immune checkpoint inhibitors; LVEF, left ventricular ejection fraction.

The therapeutic approach is not yet established. It is recommended to permanently discontinue therapy with any evidence of myocardial involvement regardless of severity (Figure 3).^152^ The standard therapy consists of initial treatment with high-dose corticosteroids (e.g. prednisone 1–2 mg/kg/day).^150^,^152^ Plasmapheresis and anti-thymocyte globulin should be considered in severe cases or with poor response to corticosteroids.^152^

## FUTURE PERSPECTIVE

The rapidly expanding repertoire of cancer therapies has revolutionized the field of oncology and led to significant reduction in cancer mortality in the last decades.^153^ The unexpected sequela of this success is the high rate of detrimental effects of both novel and traditional oncology drugs on the heart. Unfortunately, the precise mechanism for most toxic cardiomyopathies remains elusive; preventive and protective strategies are limited, and the cardiotoxic effect of most drugs is patient-specific and has limited predictability.^69^ A crucial hurdle for overcoming the abovementioned challenges is the lack of human models for evaluating cancer therapy cardiotoxicity. Recently, *in vitro* studies have shown the ability of human induced pluripotent stem cell-derived cardiomyocytes (hiPSC-CMs) to recapitulate the cardiotoxic effect of several oncological drugs.^154^–^156^ The initial studies demonstrated that the hiPSCs could mirror the cardiotoxic effects of anthracyclines,^155^–^158^ HER2-targeted therapies,^159^ and tyrosine kinase inhibitors.^154^ Importantly, initial studies suggest that this methodology may allow predicting patient-specific response to chemotherapeutic agents such as doxorubicin. The studied hiPSC-CMs from patients who experienced cardiotoxicity had increased ROS production and increased cell death.^155^ Furthermore, the hiPSC methodology may help in identifying novel mechanisms and therapeutic targets associated with chemotherapeutics cardiotoxicity. One such example was shown by Zhao et al. demonstrating that doxorubicin upregulates the expression of death receptors such as DR4, DR5, and TNF-related apoptosis-inducing ligand.^157^ Nevertheless, these models are highly limited due to the relative immaturity of the *in vitro* differentiated cardiomyocytes, the absence of the native multicellular interactions of cardiomyocytes with key supporting cells (e.g. endothelial cells and cardiac fibroblasts) commonly vulnerable to cardiotoxic effects, and the lack of the *in vivo* environment when simulating drug pharmacokinetics and long-term drug effects. Finally, the initial studies with the hiPSC-CMs platform illustrate the potential of this strategy but should be confirmed in a prospective study.

## SUMMARY

Cancer chemotherapy-related cardiac toxicity is an evolving field, so far, failing to catch up with the rapidly evolving field of cancer therapeutics. We currently have limited capability for conducting precise risk stratification, for tailoring the specific drug within a class to a specific patient, for monitoring drug-specific adverse events, and for supporting our patients with effective primary and secondary prevention measures. Further studies as well as novel technological platforms should aim at addressing these problems. In addition, the development of new chemotherapeutics should include a thorough evaluation of the cardiotoxic potential of the drug, using *in vitro* and animal models and meticulous protocols for detecting cardiac damage during the conduction of clinical trials. For now, a key to achieving successful cancer chemotherapy is viable discussion and partnership between cardiologists and oncologists, aiming together to eradicate cancer while preventing cardiotoxic sequelae.

## Abbreviations

ACEi: angiotensin-converting enzyme inhibitor
AUC: area under the curve
BNP: brain natriuretic peptide
BTZ: bortezomib
CFZ: carfilzomib
CM: cardiomyocytes
CMR: cardiac magnetic resonance
CTLA4: cytotoxic T-lymphocyte associated antigen 4
CTRCD: cancer therapeutics-related cardiac dysfunction
DOX: doxorubicin
DRZ: dexrazoxane
EMB: endomyocardial biopsy
FDA: Food and Drug Administration
GLS: global longitudinal strain
HER2: human epidermal growth factor receptor 2
hiPSC: human induced pluripotent stem cell
LV: left ventricular
LVEF: left ventricular ejection fraction
MUGA: equilibrium radionuclide angiography
NT-proBNP: N-terminal pro-brain natriuretic peptide
PD1: programmed death 1
PI: proteasome inhibitor
PLD: pegylated liposomal doxorubicin
ROS: reactive oxygen species
TnI: troponin I
VEGF: vascular endothelial growth factor.
